# PKR is not obligatory for high-fat diet-induced obesity and its associated metabolic and inflammatory complications

**DOI:** 10.1038/ncomms10626

**Published:** 2016-02-03

**Authors:** G. I. Lancaster, H. L. Kammoun, M. J. Kraakman, G. M. Kowalski, C. R. Bruce, M. A. Febbraio

**Affiliations:** 1 Cellular and Molecular Metabolism Laboratory, BakerIDI Heart and Diabetes Institute, Melbourne, Victoria 3004, Australia; 2 School of Exercise and Nutritional Sciences, Deakin University, Burwood, Victoria 3125, Australia; 3 Division of Diabetes and Metabolism, Garvan Institute of Medical Research, Darlinghurst, New South Wales 2010, Australia

## Abstract

Protein kinase R (PKR) has previously been suggested to mediate many of the deleterious consequences of a high-fat diet (HFD). However, previous studies have observed substantial phenotypic variability when examining the metabolic consequences of PKR deletion. Accordingly, herein, we have re-examined the role of PKR in the development of obesity and its associated metabolic complications *in vivo* as well as its putative lipid-sensing role *in vitro*. Here we show that the deletion of PKR does not affect HFD-induced obesity, hepatic steatosis or glucose metabolism, and only modestly affects adipose tissue inflammation. Treatment with the saturated fatty acid palmitate *in vitro* induced comparable levels of inflammation in WT and PKR KO macrophages, demonstrating that PKR is not necessary for the sensing of pro-inflammatory lipids. These results challenge the proposed role for PKR in obesity, its associated metabolic complications and its role in lipid-induced inflammation.

## Abstract (web summary)

Protein kinase R (PKR) has been suggested to act as a mediator of ER stress and inflammation in obesity. Here, Lancaster *et al*. find that genetic loss of PKR does not alter the development of obesity, and suggest that the use of littermate controls may explain differences in mouse knockout phenotypes.

## Graphical abstract

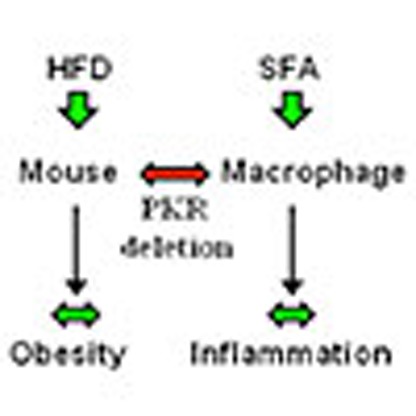

Obesity is associated with a wide array of pathologies, collectively known as metabolic diseases, which have significant morbidity and mortality. Work over the last two decades has established that chronic, low-grade inflammation is an important link between obesity and the development of metabolic diseases^1^,^2^,^3^. For example, obesity-induced inflammation within specific tissues contributes to the development of insulin resistance and type 2 diabetes^2^, hepatocellular carcinoma^4^, atherosclerosis^5^ and contributes to altered feeding behaviour, obesity and hypertension^6^,^7^.

Obesity-induced adipose tissue inflammation has been extensively characterized (for review see refs 1, 2) and plays a key role in the development of impaired glucose metabolism and insulin resistance, important developmental stages in the progression to type 2 diabetes. The development of obesity initiates a transformation of the immune cell profile within the adipose tissue, characterized by a reduction in the numbers of anti-inflammatory immune cells^8^,^9^,^10^,^11^, the recruitment of pro-inflammatory immune cells^12^,^13^,^14^,^15^,^16^ and the release of pro-inflammatory cytokines such as tumour necrosis factor α (TNFα), interleukin-1β (IL-1β), IL-6 and interferon γ. Classically, it is considered that specific pro-inflammatory lipids activate the nuclear factor κB (NF-κB) and JNK signalling pathways within macrophages, adipose tissue and the liver leading to pro-inflammatory cytokine production, impaired insulin signalling via insulin receptor substrate 1 serine phosphorylation, impaired glucose metabolism and insulin resistance^2^,^3^. More recent work points to adipose tissue inflammation-induced lipolysis as being a central mediator of elevated hepatic glucose production and obesity-induced hyperglycaemia^17^. Given the importance of inflammation in the development of impaired glucose metabolism and insulin resistance, considerable effort has been made in understanding how the nutrient-surplus associated with obesity, in particular chronically elevated lipids such as fatty acids, activates pro-inflammatory signalling pathways such as NF-κB and JNK. In this regard, several processes have been identified that couple the ‘sensing' of changes in intra- and extra-cellular lipid levels to the activation of inflammatory signalling pathways. These include c-Src activation and clustering within membrane subdomains^18^, endoplasmic reticulum stress^19^,^20^, the nucleotide-binding domain, leucine-rich repeats containing family, pyrin domain-containing-3 (NLRP3) inflammasome^21^, Toll-like receptor 4 (refs 22, 23) and protein kinase R (PKR)^24^.

PKR is best known for its function in the innate immune system where it plays a key role in the resistance to viral infection^25^. However, recent studies have shown that PKR is activated by chronically elevated levels of specific nutrients^24^ and that the deletion of PKR has a number of effects on whole-body metabolism including protection against high-fat diet (HFD)-induced obesity, glucose intolerance, insulin resistance and inflammation^24^,^26^,^27^. More recent studies have identified binding of small nucleolar RNAs to PKR following exposure to the saturated fatty acid palmitate, and have suggested that PKR is an indirect sensor of nutrient surplus^28^. However, although previous studies have concluded that the deletion of PKR prevents the deleterious consequences of a HFD, there is nonetheless considerable phenotypic variation observed in the previous studies that have examined the metabolic consequences of PKR deletion^24^,^26^,^27^. For example, although initial studies showed that PKR knockout (KO) mice were profoundly protected from HFD-induced obesity^24^,^27^, more recent studies show no protection from hyperphagia-induced obesity when PKR KO mice are crossed to the genetically obese *ob*/*ob* background^26^.

Given the potential importance of PKR as a sensor of chronic nutrient excess and in mediating the deleterious metabolic consequences of high fat (HF) feeding, we sought to clarify the previously described role of PKR in the development of HFD-induced obesity, impaired glucose metabolism and inflammation. Here we show that the deletion of PKR has no effect on HFD-induced obesity or the development of impaired glucose metabolism, and has only a modest effect on adipose tissue inflammation.

## Results

### Study in non-littermate PKR KO and wild-type (WT) mice

The PKR KO mice used herein have been backcrossed to the C57Bl/6 background (greater than 20 generations)^29^ and have been used in numerous previous studies to examine the role of PKR in multiple experimental settings^30^,^31^,^32^. In preliminary experiments examining the role of PKR in metabolism, we compared PKR KO mice with non-littermate WT C57Bl/6 mice. Following 10 weeks of a HFD, PKR KO mice had a moderately exacerbated obesity phenotype compared with their WT counterparts (fat mass=17.9±0.9 g versus 13.1±2.2 g; *P*<0.001; for PKR KO, *n*=7; and WT, *n*=8, respectively). These results were opposite to two previous studies that demonstrate protection from HFD-induced obesity in PKR KO mice^24^,^27^. In attempting to reconcile these discrepant findings, we considered that the animal breeding strategy employed may have contributed to the divergent results of our study and those published previously^24^,^27^. Specifically, we obtained PKR KO mice from crosses of mice homozygous for the mutated *Eif2ak2* allele and compared these with C57Bl/6 WT mice. This may have potentially impacted on the results of our study in several ways: for example, differences in the gut microbiome, which are influenced by the parental lineage and husbandry conditions within animal houses, can profoundly affect metabolic phenotypes, and genetic/epigenetic differences that may have arisen between the two independently maintained lines of mice. Accordingly, to control for potential genetic or microbiome differences, we conducted extensive experiments using WT and PKR KO littermate control mice obtained from crosses of mice heterozygous for the mutated *Eif2ak2* allele. All the data contained herein were generated using WT and PKR KO littermate control mice obtained from crosses of mice heterozygous for the mutated *Eaf2ak2* allele.

### Loss of PKR does not prevent the development of obesity

**Figure 1:**
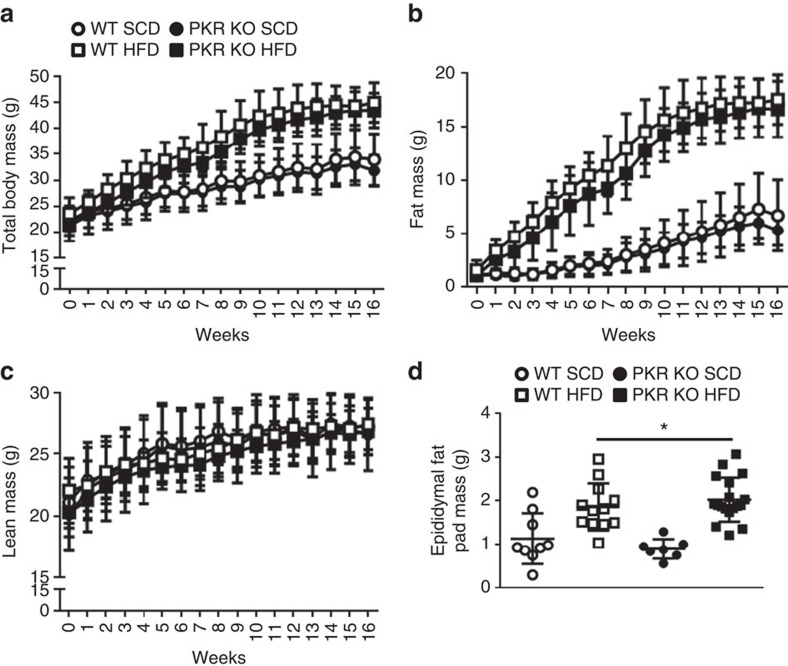
Loss of PKR does not prevent the development of obesity. Total body mass (**a**), fat mass (**b**), lean mean (**c**) and epididymal adipose tissue mass (**d**) in WT and PKR KO mice fed either a HFD or SCD for 16 weeks. *N*s are 9, 8, 12 and 18 for WT SCD, PKR KO SCD, WT HFD and PKR KO HFD, respectively. Data are presented as the mean±standard deviation (s.d.). *P<0.001, two-way ANOVA, main effect of diet between SCD and HFD.

WT and PKR KO mice were fed with either a standard chow diet (SCD; 12% kJ from fat) or HFD (43% kJ from fat) for a period of 16 weeks commencing at 6 weeks of age. Compared with standard chow (SC)-fed mice, HF-fed mice gained significantly more weight over the course of the dietary period (Fig. 1a), an effect that was attributable to an increase in fat mass (Fig. 1b,c). However, the loss of PKR did not affect the development of obesity whatsoever (Fig. 1a,b,d).

### Loss of PKR does not improve glucose metabolism

**Figure 2:**
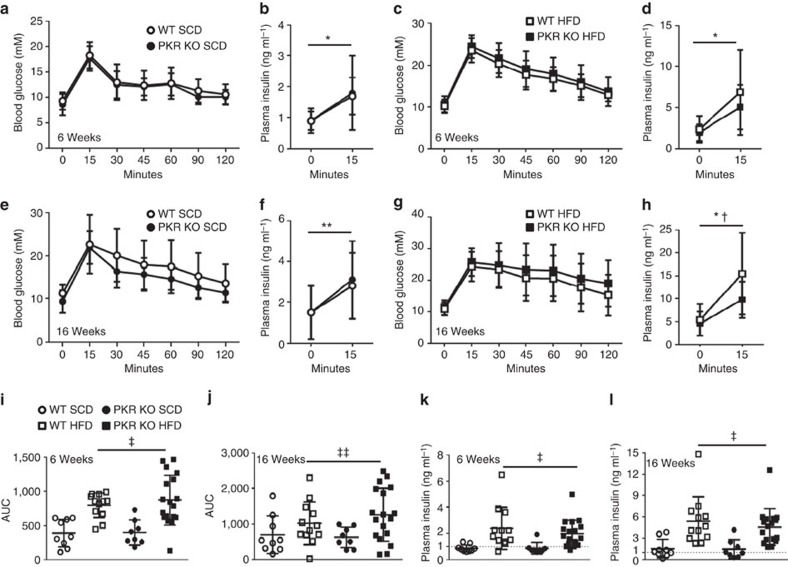
Loss of PKR does not improve glucose metabolism in HF-fed mice. Glucose tolerance as assessed by OGTT (**a**,**c**,**e**,**g**) and plasma insulin during the OGTT (**b**,**d**,**f**,**h**), in WT and PKR KO mice after 6 (**a**–**d**) and 16 (**e**–**h**) weeks of either a SCD (**a**,**b**,**e**,**f**) or HFD (**c**,**d**,**g**,**h**). The area under the curve for the OGTT in all groups at 6 (**i**) and 16 weeks (**j**). Fasting plasma insulin concentrations in all groups at 6 (**k**) and 16 weeks (**l**). Except for plasma insulin following 16 weeks of HFD in PKR KO mice where *N*=17, *N*s are 9, 8, 12 and 18 for WT SCD, PKR KO SCD, WT HFD and PKR KO HFD, respectively. Data are presented as the mean±standard deviation (s.d.). *P<0.001, two-way ANOVA, main effect for time between 0 and 15 min. **P<0.01, two-way ANOVA, main effect for time between 0 and 15 min. †*P*=0.018, two-way ANOVA, main effect for genotype between WT and PKR KO. ‡*P*<0.001, two-way ANOVA, main effect of diet between SCD and HFD. ‡‡*P*=0.01, two-way ANOVA, main effect of diet between SCD and HFD.

Although our PKR KO mice did not display an obesity phenotype, it is not uncommon for glucose intolerance and/or insulin resistance to be uncoupled from obesity in genetically modified mice. Accordingly, we performed oral glucose tolerance tests (OGTTs) in WT and PKR KO mice following 6 and 16 weeks of dietary intervention, time points that reflect differing levels of obesity (Fig. 1a,b). Following 6 weeks of the dietary period, mice fed the HFD were significantly glucose intolerant compared with those fed the SCD (Fig. 2a,c,i). Both fasting plasma insulin concentrations and the increase in plasma insulin 15 min following the administration of the glucose bolus were exacerbated in HF-fed mice (Fig. 2b,d,k), indicative of systemic insulin resistance. These data confirm the efficacy of the HFD regimen we employed to induce impaired glucose tolerance and insulin resistance. The loss of PKR, however, neither affected glucose metabolism nor plasma insulin levels after 6 weeks of either SCD or HFD (Fig. 2a–d,i,k). Glucose tolerance, fasting plasma insulin and plasma insulin during the OGTT were further worsened following 16 weeks of HFD (Fig. 2e–h,j,l). Although the loss of PKR did not affect glucose tolerance following 16 weeks of either the SCD (Fig. 2e) or HFD (Fig. 2g), we did observe significantly lower plasma insulin levels in PKR KO mice compared with their WT counterparts following 16 weeks of HFD (Fig. 2h; genotype main effect, *P*=0.018). This effect was primarily driven by the plasma insulin concentrations during the OGTT (15 min time point) as no significant difference was observed between genotypes in fasting insulin concentrations (Fig. 2l).

### Loss of PKR does not improve hepatic steatosis

**Figure 3:**
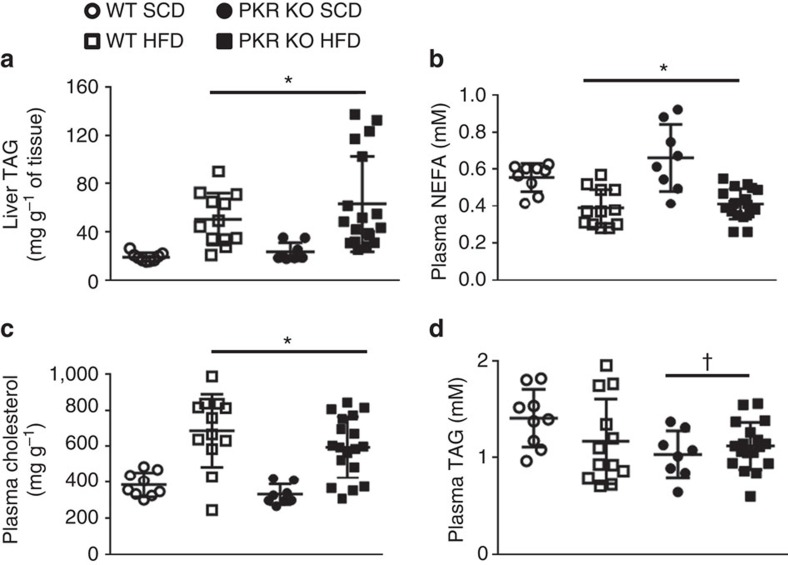
Loss of PKR does not improve hepatic steatosis in HF-fed mice but decreases plasma TAG. Liver TAG (**a**), plasma NEFA (**b**), plasma cholesterol (**c**) and plasma TAG (**d**) in WT and PKR KO mice fed either a HFD or SCD for 16 weeks. *N*s are 9, 8, 12 and 18 for WT SCD, PKR KO SCD, WT HFD and PKR KO HFD, respectively. Data are presented as the mean±standard deviation (s.d.). *P<0.001, two-way ANOVA, main effect for diet between SCD and HFD. †*P*=0.034, two-way ANOVA, main effect for genotype between WT and PKR KO.

Hepatic steatosis is a hallmark of obesity and deletion of PKR was previously shown to prevent HFD-induced hepatic lipid accumulation^24^. As expected, we observed a significant increase in hepatic triacylglycerol (TAG) levels following the HFD (Fig. 3a). However, loss of PKR had no effect on TAG accumulation (Fig. 3a). We observed a significant decrease in fasting plasma-free fatty acids (Fig. 3b) and an increase in fasting plasma cholesterol levels (Fig. 3c) following 16 weeks of HFD, neither of which were affected by the loss of PKR (Fig. 3b,c). Although plasma TAG levels were unaffected by the HFD, we did, however, observe a significant decrease in plasma TAG levels in PKR KO mice compared with their WT counterparts (Fig. 3d).

### PKR deletion moderately affects adipose tissue inflammation

**Figure 4:**
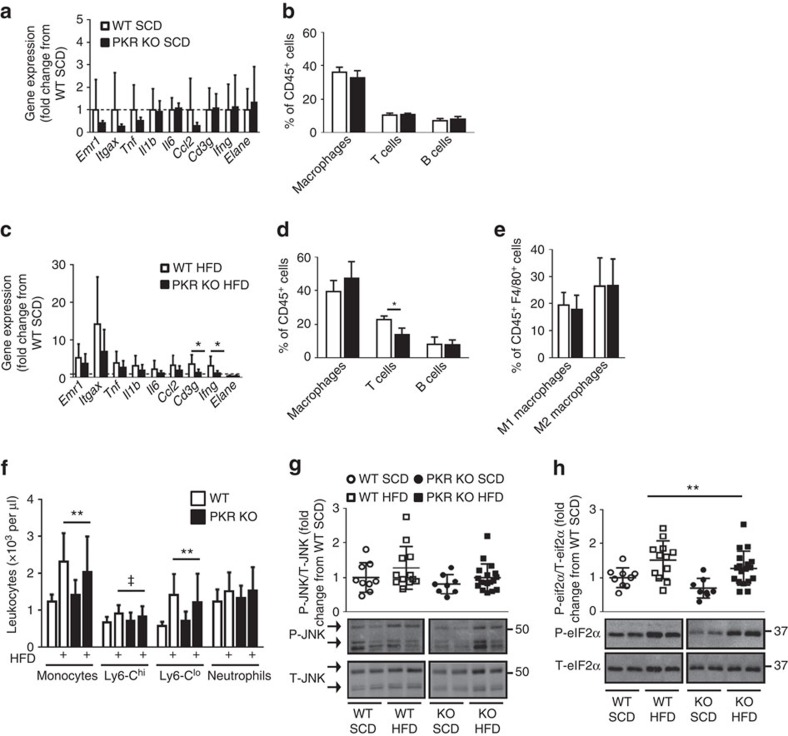
Loss of PKR does not prevent HFD-induced adipose tissue macrophage recruitment, but ameliorates T-cell recruitment. Inflammatory gene expression (**a**,**c**), percentages of specific immune cell populations (**b**,**d**,**e**), circulating monocyte and neutrophil numbers (**f**), phosphorylation of JNK (**g**) and the phosphorylation of eIF2α (**h**) within WAT in WT and PKR KO mice fed either a HFD (**c**–**h**) or SCD (**a**,**b**,**f**–**h**) for 16 weeks. *N*s for WT SCD, PKR KO SCD, WT HFD and PKR KO HFD, respectively, for each figure are as follows: (**a**) 8–9 and 7; (**b**) 4 and 4; (**c**) 11–12 and 13–15; (**d**) 5 and 8; (**e**) 5 and 4; (**f**) 9, 8, 12 and 18; (**g**) 9, 8, 12 and 18; (**h**) 9, 8, 12 and 18. Data are presented as the mean±standard deviation (s.d.). *P<0.05, independent samples *t*-test. **P<0.001, two-way ANOVA, main effect for diet between SCD and HFD. ‡*P*<0.05, two-way ANOVA, main effect of diet between SCD and HFD.

Adipose tissue immune cell recruitment and inflammation are a hallmark of obesity and are thought to play a critical role in the development of glucose intolerance and insulin resistance. The loss of PKR has previously been reported to reduce adipose tissue inflammation^24^. Although we did not observe a protective effect of PKR deletion on either the development of obesity or glucose intolerance, we nonetheless sought to determine whether the loss of PKR could prevent adipose tissue inflammation, as we recently demonstrated that adipose tissue macrophage recruitment due to HF feeding could be prevented, in the absence of whole-body changes in insulin sensitivity^33^. As expected, mice fed a HFD displayed many of the hallmarks of adipose tissue inflammation such as macrophage and T-cell recruitment and an increase in the expression of pro-inflammatory cytokine expression (Fig. 4a–d and Supplementary Figs 1–2). Loss of PKR did not prevent macrophage recruitment into adipose tissue (Fig. 4c,d), affect macrophage polarization state (Fig. 4e) or the expression of pro-inflammatory cytokines including *Tnf*, *Il1b* and *Il6* (Fig. 4c). A feed forward loop has recently been described in which obesity promotes monocytosis and neutrophilia, contributing to enhanced adipose tissue macrophage accumulation^34^. Although we observed monocytosis following 16 weeks of HFD, the loss of PKR was without effect on HFD-induced monocytosis (Fig. 4f and Supplementary Fig. 3). Neutrophil numbers were not affected by either the HFD or the loss of PKR (Fig. 4f). However, the loss of PKR did prevent the recruitment of T cells into the adipose tissue following 16 weeks of HFD, as demonstrated by both decreased *Cd3g* and *Ifng* gene expression (Fig. 4c) and reduced numbers of TCRβ^+^ T cells within the adipose tissue as determined by flow cytometry (Fig. 4d).

Activation of JNK within the adipose tissue is proposed to play an important role in the development of both inflammation and insulin resistance. Although we and others have previously observed increased JNK activation in the adipose tissue of HF-fed mice^35^,^36^, in the present study we were unable to observe any significant dietary effect on JNK phosphorylation status (Fig. 4g and Supplementary Fig. 4) and the loss of PKR was also without effect on JNK phosphorylation status (Fig. 4g and Supplementary Fig. 4). It has previously been shown that obesity activates PKR^24^ and that eIF2α, PKR's canonical substrate, is also activated by obesity. Consistent with the previous literature^20^, we observed a significant increase in the phosphorylation of eIF2α in the adipose tissue of HF-fed mice (Fig. 4h and Supplementary Fig. 4). Of note, we observed a borderline significant decrease in phosphorylated eIF2α levels in PKR KO animals (two-way analysis of variance (ANOVA), genotype main effect *P*=0.05), suggesting that PKR indeed contributes to eIF2α phosphorylation status in adipose tissue, irrespective of dietary status (Fig. 4h and Supplementary Fig. 4). However, and importantly, the relative increase in eIF2α phosphorylation following the HFD was nearly identical in both WT and PKR KO mice (Fig. 4h and Supplementary Fig. 4), demonstrating that PKR plays a minimal role in the regulation of eIF2α phosphorylation in adipose tissue following HF feeding. One of eIF2α's additional three upstream kinases, such as PKR-like endoplasmic reticulum kinase, which has previously been shown to be activated in obese adipose tissue^20^, is likely responsible for the increased eIF2α phosphorylation we observed in adipose tissue from HF-fed WT and PKR KO mice.

### PKR deletion does not prevent lipid-induced inflammation

**Figure 5:**
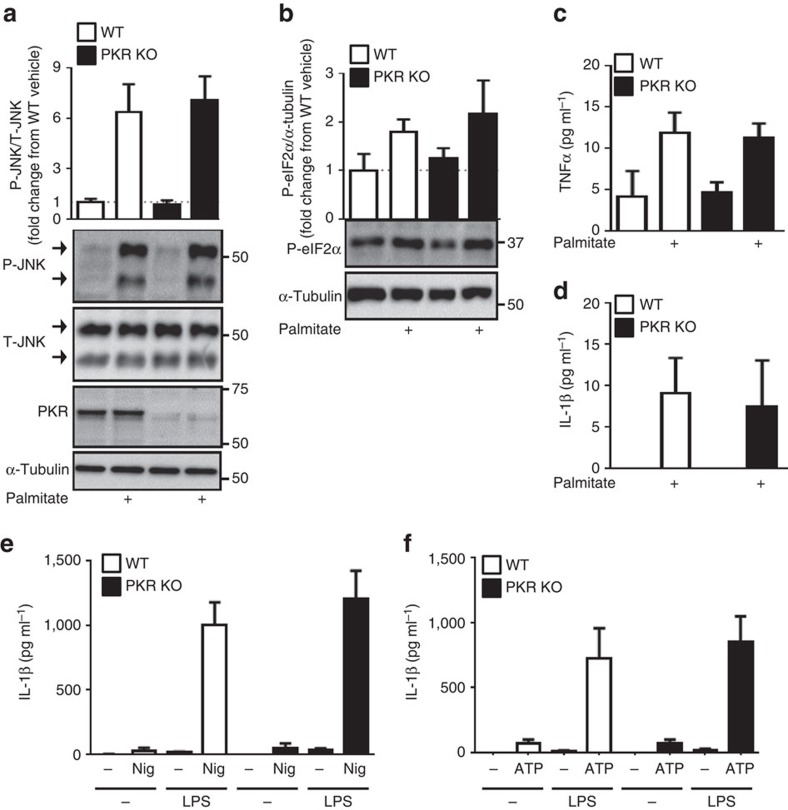
Loss of PKR *in vitro* does not prevent saturated fatty acid-induced inflammation or inflammasome activation. Phosphorylation of JNK (**a**), eIF2α (**b**), secretion of TNFα (**c**) and the secretion of IL-1β (**d**) in BMDM from WT and PKR KO mice treated with 1 mM palmitate or vehicle for 4 h (**a**,**b**) or 8 h (**c**,**d**). (**e**,**f**) The secretion of IL-1β in WT and PKR KO BMDM following treatment with either Nigericin (Nig; **e**) or ATP (**f**). All the data represent three independent mice per genotype with BMDM from each mouse comprised of three technical replicates. Data are presented as the mean±standard deviation (s.d.).

As discussed above, PKR is proposed to be a central component of a complex that responds to nutrients, promoting inflammation in times of nutrient excess^24^. Long-chain saturated free fatty acids (lcSFA) exert pro-inflammatory actions via number of mechanisms, and PKR has previously been shown to be activated by lcSFA and deletion of PKR prevented lcSFA-induced inflammation^24^. To test the role of PKR in lcSFA-induced inflammation *in vitro*, we treated primary bone marrow-derived macrophages (BMDM) with the lcSFA palmitate for 4 h and assessed the phosphorylation status of JNK. Although JNK phosphorylation was markedly increased following treatment with palmitate, the loss of PKR was without effect on palmitate-induced JNK phosphorylation (Fig. 5a and Supplementary Fig. 5). Likewise, palmitate increased eIF2α phosphorylation in WT and PKR KO BMDM to a similar extent (Fig. 5b and Supplementary Fig. 5). In a separate set of experiments, we treated WT and PKR KO BMDM with palmitate for 8 h and assessed the amount of TNFα and IL-1β released into the cell culture media. Palmitate treatment increased the concentrations of TNFα and IL-1β in the media, with WT and PKR KO BMDM releasing comparable amounts of TNFα and IL-1β (Fig. 5c,d). Inflammasomes are multimeric protein complexes that control the activation of precursor IL-1β (ref. 37). The role of PKR in inflammasome activation is controversial, with evidence that loss of PKR prevents inflammasome activation^38^, or has no effect on inflammasome activation^39^. Given the important role of the NLRP3 inflammasome in mediating obesity-induced inflammation, impaired glucose metabolism and insulin resistance^21^, we examined the role of PKR in NLRP3 inflammasome activation. Following 3 h of priming with lipopolysaccharide (LPS), we treated WT and PKR KO BMDM with the NLRP3 inflammasome activators nigericin and ATP. As expected, LPS ‘primed' BMDM produced significantly more IL-1β when treated with either nigericin (Fig. 5e) or ATP (Fig. 5f); however, we observed no differences in IL-1β release between WT and PKR KO BMDM.

### Poly(I:C) does not impair glucose tolerance

**Figure 6:**
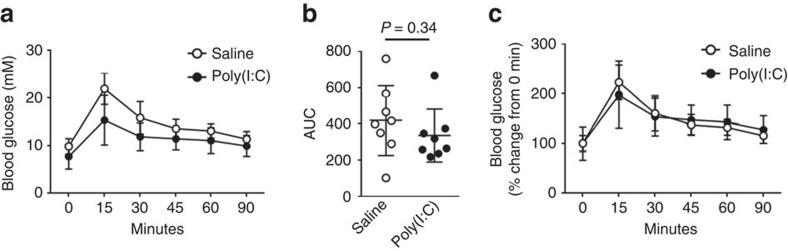
Injection of Poly(I:C) does not impair glucose metabolism. Glucose tolerance as assessed by OGTT in C57Bl6/J mice administered with either sterile saline or 10 mg kg^−1^ Poly(I:C) 6 h before OGTT (**a**–**c**). *N*s are 8 for both groups. Data are presented as the mean±standard deviation (s.d.). An independent samples *t*-test was used to compare the area under the curve between the two groups.

In a final set of experiments, we determined whether the injection of polyinosinic:polycytidylic acid (Poly(I:C)), a synthetic analogue of double-stranded RNA, and hence an activator of PKR, could impair glucose tolerance. Based on previous studies that have examined the cytokine^40^, physiological^41^ and behavioural^41^ responses of mice to Poly(I:C), we injected C57Bl6/J mice with 10 mg kg^−1^ of Poly(I:C) or a sterile saline control 6 h before the assessment of glucose tolerance by OGTT. As shown in Fig. 6a–c, Poly(I:C) did not impair glucose tolerance relative to sterile saline.

## Discussion

The results of the present study show that the deletion of PKR has no effect on HFD-induced obesity or the development of impaired glucose metabolism. Three previous studies from two independent laboratories have examined the effects of the loss of PKR on metabolism *in vivo*^24^,^26^,^27^, and, although each of these studies has concluded that the deletion of PKR protects against the deleterious effects of a HFD, there are nonetheless substantial differences in the phenotypes reported in these previous studies, highlighting that the role of PKR *in vivo* in the context of metabolic disease remains to be fully clarified. In the original report by Nakamura *et al*. on the role of PKR in metabolism, deletion of PKR largely prevented HFD-induced obesity and decreased fasting plasma insulin and fasting blood glucose concentrations^24^. After 6 weeks of HFD, a time point at which no differences in bodyweight were observed, PKR KO mice had improved glucose clearance during a GTT compared with WT mice^24^. Although PKR KO mice also had improved glucose tolerance following 14 weeks of HFD, it is important to note that WT mice were ∼30% heavier than PKR KO mice at this time point. As the bolus of glucose administered during the GTT was relative to bodyweight, the putative improvement in glucose tolerance may reflect the commensurately lower bolus of glucose administered to the PKR KO mice^24^. Similarly, although it is stated that PKR KO mice had improved insulin tolerance, the difference between the WT and PKR KO mice is primarily accounted for by alterations in basal glucose levels, with the hypoglycaemic response to insulin administration similar between WT and PKR KO mice^24^; although it should be noted that PKR KO mice did have improved insulin signalling responses, suggesting that at least to some extent insulin action was enhanced in PKR KO mice compared with WT controls^24^. PKR KO mice also had reduced adipose tissue inflammation as indicated by the reduced gene expression of a number of pro-inflammatory cytokines as well as markers of macrophage infiltration^24^. Finally, when fed a SCD, no phenotypic differences were observed between WT and PKR KO mice^24^.

In the study by Carvalho-Filho *et al*.^27^ PKR KO mice, regardless of being fed either a SCD or HFD, had fasting blood glucose concentrations of ∼3 mM (50–60 mg dl^−1^), compared with ∼8 mM (150 mg dl^−1^) for the WT mice. In mice, a blood glucose concentration of 3 mM represents hypoglycaemia, indicating that the loss of PKR in this study resulted in a profound disruption to the control of glucose metabolism. Although the PKR KO mice in the Carvalho-Filho *et al*. study had markedly improved glucose tolerance following a HFD, it is again important to note that mice were given a bolus of glucose that was body weight dependent. Because HF-fed WT mice weighed ∼50% more than PKR KO mice, it is possible that the improved glucose tolerance observed in PKR KO mice is mediated by the reduced bolus of glucose that PKR KO mice received. However, it should be noted that Carvalho-Filho *et al*. show an increase in the glucose infusion rate required to maintain euglycaemia during a hyperinsulinaemic-euglycaemic clamp, indicative of improved insulin sensitivity. Finally, in contrast to the original report of Nakamura *et al*.^24^ Carvalho-Filho *et al*. show improved glucose tolerance, and, as noted above, reduced fasting blood glucose levels, in PKR KO mice fed a SCD.

In a recent study by Nakamura *et al*.^26^ PKR KO mice were backcrossed to the C57Bl/6 background before being intercrossed with mice heterozygous for the *ob* (leptin) mutation to generate PKR KO-*ob*/*ob* mice. In contrast with the previously reported protective effect of PKR deletion on HFD-induced obesity^24^,^27^, *ob*/*ob*-PKR KO mice gained identical amounts of weight compared with *ob*/*ob* control mice^26^. Although fasting blood glucose levels were reduced in *ob*/*ob*-PKR KO mice, fasting plasma insulin levels were unaltered between *ob*/*ob*-PKR KO and *ob*/*ob* control mice^26^. Furthermore, although it was concluded that PKR KO-*ob*/*ob* mice have improved glucose clearance and insulin sensitivity, as assessed by glucose and insulin tolerance tests, respectively, these differences are primarily due to differences in fasting blood glucose levels^26^. Based on the data presented by Nakamura *et al*.^26^ the glucose clearance, as inferred from the area under the curve, and insulin sensitivity, as inferred from the absolute decrease in the blood glucose concentration following insulin administration, are similar in PKR KO-*ob*/*ob* mice and *ob*/*ob* control mice^26^. These data suggest that when crossed to the *ob*/*ob* background, the deletion of PKR has little effect on obesity or insulin sensitivity.

Finally, in agreement with the results reported herein, in a study examining the contribution of PKR to the decreased food intake, reduced weight gain and bodyweight loss induced by the ribotoxic mycotoxin, trichothecene deoxynivalenol, it was shown that WT and PKR KO mice not receiving deoxynivalenol gained similar amounts of weight when fed a HFD for 15 weeks^42^.

It is important to note that two distinct lines of PKR KO mice have been generated and the studies described above have used one or the other of these PKR KO mouse lines. The originally reported PKR KO mouse line was generated by the disruption of exons 2 and 3 of *Eif2ak2* (ref. 29) and the second by disruption of exon 12 of *Eif2ak2* (ref. 43). Henceforth, for convenience, we refer to these two lines as N-PKR KO^29^ and C-PKR KO^43^. In the present study, we used the N-PKR KO mice on a C57Bl/6 genetic background, which contrasts with Nakamura *et al*.^24^ who used the C-PKR KO mice on a mixed 129Sv x BALB/c background, and these differences may account for the phenotypic differences observed between the two studies. Indeed, differences have been reported in the signalling responses of cells isolated from C-PKR KO and N-PKR KO mice to specific stimuli^29^,^43^. However, the study by Carvalho-Filho *et al*.^27^ used the same N-PKR KO mouse line on the C57Bl/6 genetic background as used herein. It is difficult to reconcile the markedly different phenotypes that were observed between Carvalho-Filho *et al*.^27^ and that reported herein, although different husbandry conditions between animal facilities may potentially explain some of the differences. Finally, Flannery *et al*.^42^ also used the N-PKR KO line on the C57Bl/6 genetic background, as used herein, and reported a very similar effect of PKR deletion on HFD-induced weight gain to that observed in our study, that is, no effect.

It is important to highlight that while we conclude that the deletion of PKR does not affect HFD-induced obesity or glucose metabolism, a conclusion that is at odds with the proposed role of PKR in metabolic homeostasis, there are nonetheless several aspects of the phenotype that we describe that are in agreement with previous reports that claim a protective effect of PKR deletion. Consistent with Nakamura *et al*.^26^ who examined the effect of PKR deletion in *ob*/*ob* mice, we see no effect of PKR deletion on the development of obesity, fasting plasma insulin levels or glucose tolerance. Furthermore, both as reported by Nakamura *et al*.^26^ and herein, PKR KO mice had a decrease in plasma TAG levels. Consistent with the original report by Nakamura *et al*.^24^ we see a modest reduction in adipose tissue inflammation, albeit we did not observe reductions in macrophage infiltration or the expression of classical pro-inflammatory cytokines such as *Tnfa* and *Il1b*. In addition, both in the present study and that by Nakamura *et al*.^24^ PKR KO mice fed a SCD were indistinguishable from their WT counterparts. Finally, although we did not observe an improvement in glucose tolerance following 16 weeks of HFD in PKR KO mice, plasma insulin levels during the OGTT were significantly lower, indicative of increased insulin sensitivity and consistent with the original report by Nakamura *et al*.^24^ Collectively, the results of previous studies examining the effect of PKR deletion on obesity, glucose metabolism and insulin resistance demonstrate substantial phenotypic variability and, in some instances, relatively modest effects. Our results demonstrate that the deletion of PKR has no role in HFD-induced obesity and, at most, only a minor effect on its associated complications such as adipose tissue inflammation and impaired glucose metabolism. In light of the central role that PKR is postulated to play in mediating the deleterious effect of a HFD and obesity, the contribution of PKR to the development of obesity and its associated complications needs further research.

As the PKR KO mice we used in our studies were extensively backcrossed to the C57Bl/6 background, in our initial preliminary studies we compared PKR KO mice with C57Bl/6 WT controls. Although this is a common experimental approach, it is nonetheless subject to potential sources of bias, which may confound the interpretation of experimental results. In our initial study, PKR KO mice were transferred to our institution at ∼4 weeks of age following breeding at a separate institution. At ∼10 weeks of age, these PKR KO mice and C57Bl/6 WT mice obtained from a colony maintained within our institution commenced HF feeding. Accordingly, a number of factors may have influenced the results of this study, such as genetic/epigenetic differences, which may have arisen between the independently maintained C57Bl/6 WT and PKR KO mouse colonies and differences in the gut microbiome. Accordingly, and because the results of our initial study contradicted two previously published reports, we were concerned that the phenotype we observed may have been the result of factors independent of the deletion of PKR. To control for potential genetic, microbiome or other differences, we generated PKR KO and WT littermates by crossing mice heterozygous for the mutated *Eif2ak2* allele. The differences that we observed between our initial study and the subsequent study using WT and PKR KO littermate controls underscores the importance of using littermate control mice in all studies examining altered metabolic homeostasis.

It was previously shown that murine embryonic fibroblasts (MEFs) lacking PKR were resistant to the pro-inflammatory effects of the saturated fatty acid palmitate^24^. Given that we did not observe any effect *in vivo* of PKR deletion on HFD-induced obesity or glucose metabolism, we next decided to test the proposed lipid sensing function of PKR by determining if the loss of PKR compromised the ability of macrophages to respond to pro-inflammatory saturated fatty acids. Unlike MEFs, macrophages are important pro-inflammatory cells *in vivo* in the context of obesity where they are proposed to respond to pro-inflammatory lipids. Using primary macrophages, we show that the deletion of PKR did not prevent the ability of palmitate to activate JNK or eIF2α or to stimulate the release of the pro-inflammatory cytokines TNFα or IL-1β. Although these data are consistent with the observed *in vivo* phenotype of HF-fed PKR KO mice reported herein, they are nonetheless at odds with previous findings^24^. It is possible that differences in the cell types used, primary macrophages versus MEFs, or the fact that our primary macrophages were derived from N-PKR KO mice and the MEFs used in Nakamura *et al*. were obtained from C-PKR KO mice, may account for the differences we observed in the cellular response to palmitate. Indeed, as noted above, MEFs from C-PKR KO and N-PKR KO have previously been shown to have distinct responses to pro-inflammatory stimuli^29^,^43^.

In conclusion, using a well-established and accepted mouse model of PKR deletion, we find that the loss of PKR has no effect on the ability of cells to sense pro-inflammatory lipids *in vitro*, and does not alter the development of HFD-induced obesity or glucose intolerance *in vivo*. Although these results contradict the proposed function of PKR in the regulation of metabolic homeostasis, our findings are nonetheless consistent with numerous aspects of previous studies that have examined the metabolic consequences of PKR deletion. Given the important role that PKR is proposed to play in the development of obesity and its associated metabolic consequences, the role of PKR in the control of metabolic homeostasis requires further, independent confirmation.

## Methods

### Mouse experiments

The PKR KO mice used in the present study were generated previously^29^. As discussed above, in the initial preliminary study we used PKR KO male mice obtained from crosses of mice homozygous for the mutated null *Eif2ak2* allele and compared these with age-matched C57Bl/6 WT non-littermate control male mice. All data shown in Figs 1, 2, 3, 4, 5 are from experiments conducted in PKR KO and WT littermate control mice obtained from crosses of mice heterozygous for the mutated null *Eif2ak2* allele, which were bred and housed at the Alfred Medical Research and Education Precinct Animal Centre (AMREP PAC). In the Poly(I:C) experiment described in Fig. 6, 8-week-old male C57Bl6/J mice fed a SCD diet were used. Mice were housed at 22±1 °C on a 12:12 h light/dark cycle and had *ad libitum* access to food and water. For all the *in vivo* experimental procedures described below, littermate control, male mice were fed either a SCD containing 12% of energy from fat (Meat Free Rat and Mouse Diet; Total digestible energy 14.0 MJ kg^−1^; Specialty Feeds) or a HFD containing 43% of energy from fat (SF04-001; Total digestible energy 19 MJ kg^−1^; Specialty Feeds) commencing at 6 weeks of age for a total of 16 weeks. The animals were randomly assigned to SCD or HFD groups in a non-blinded manner. All animal experiments were approved by the AMREP animal ethics committee and were performed in accordance with National Health and Medical Research Council (NHMRC) of Australia Guidelines for Animal Experimentation.

### Oral glucose tolerance tests

Following a 5-h fast, glucose tolerance was assessed by the oral administration of 2 g of glucose per kg of lean mass. Before the administration of glucose and at the indicated time points post glucose administration, blood was collected from the tip of the tail and blood glucose concentrations were determined using a glucometer (Accu-Check, Roche). Additional blood samples were collected at 0 and 15 min following glucose administration and the plasma obtained for the determination of insulin concentrations.

For the Poly(I:C) (Invivogen; LMW Poly(I:C)) experiments described in Fig. 6, 4 g of glucose per kg of lean mass was administered. Poly(I:C) reconstituted in sterile saline or the sterile saline vehicle alone were injected intraperitoneally 6 h before the OGTT. To minimize disturbances to the mice, mice were fasted 6 h before the OGTT, that is, immediately following Poly(I:C) or sterile saline injection. The OGTT was conducted as described above.

### Body composition

Mice were weighed to determine total body mass and fat and lean mass were determined using an EchoMRI 4-in-1 (Echo Medical Systems).

### Plasma insulin

Plasma insulin concentrations were determined using a mouse ultrasensitive plasma insulin ELISA (ALPCO). Where required, samples were diluted according to the manufacturer's instructions.

### WT and PKR KO primary BMDM

Bone marrow-derived macrophages were generated from bone marrow isolated from the hind limbs of 8-week-old male, PKR KO and WT littermate control mice. Cells were cultured in RPMI media containing 15% fetal bovine serum, 20% L929-cell conditioned media and 1% penicillin/streptomycin. On the day before experimentation, media were changed to RPMI containing 5% fetal bovine serum and 2% (w/v) bovine serum albumin (BSA; A6003, Sigma). Palmitate (P0500; Sigma-Aldrich) was dissolved in ethanol at 100 mM and conjugated to BSA-containing media with gentle rocking at 37 °C at a final concentration of 1 mM. The vehicle contained both BSA and ethanol. For inflammasome experiments, BMDM were primed with LPS (Invivogen) at a concentration of 100 ng ml^−1^ for 3 h before treatment with either Nigericin (10 μM) or ATP (4 mM; Sigma-Alrdich) for 0.5 h.

### TNFα and IL-1β ELISAs

Levels of TNFα (DY410) and IL-1β (DY401) in cell culture supernatants were assessed by DuoSet ELISA (R&D Systems).

### Immunoblotting

Epididymal white adipose tissue (WAT) was collected following dissection and a portion was snap frozen in liquid nitrogen. Approximately 50 mg of WAT was homogenized in lysis buffer containing HEPES (20 mM), EGTA (2 mM), β-glycerophosphate (50 mM), dithiothreitol (0.1 mM), Na_3_VO_4_ (0.1 mM), NaF (1 mM), phenylmethylsulphonyl fluoride (1 mM), protease inhibitor cocktail (Sigma; P8340) and Phosphatase Inhibitor Cocktail 2 (Sigma; P5726). Lysates were kept on ice until being spun at 16,000*g* for 20 min at 4 °C, the supernatant collected and protein concentrations determined using the BCA method (ThermoScientific). Twenty micrograms of total protein were solubilized in Laemmli buffer and heated at 95 °C for 5 min. For BMDM, cells were washed twice with ice-cold PBS and then scraped in ice-cold lysis buffer before samples were prepared as described above. Proteins were resolved by SDS–polyacrylamide gel electrophoresis, transferred to 0.2 μm nitrocellulose membranes (Bio-Rad), blocked in 5% skim milk, washed in tris-buffered saline and tween 20 (TBST) and incubated in primary antibodies overnight at 4 °C. The molecular weight ladder used was the Precision Plus Protein Standards (#161-0373; Bio-Rad). The following primary antibodies were used at 1:1,000: pJNK (Cell Signaling, 9251), tJNK (Cell Signaling, 9258), peIF2α (Cell Signaling. 3597), teIF2α (Cell Signaling, 9722), PKR (Santa Cruz, sc-6282) and α-tubulin (Sigma, T5168). Following incubation in primary antibody membranes were washed in TBST and incubated in either rabbit (NA934V, Anti-Rabbit IgG-HRP, GE Healthcare) or mouse (NXA931, Anti-Mouse IgG-HRP, GE Healthcare) secondary antibodies for 1 h at room temperature. Following a final wash, signals were detected by chemiluminescence (ThermoScientific, 34095 and 34080) and specific bands quantified using Quantity One (Bio-Rad).

### Flow cytometry

For flow cytometry, WAT was dissected and placed in PBS before being finely minced in PBS+0.1% BSA containing collagenase B (Roche). After incubation at 37 °C for 45 min, a 10 × volume of PBS+0.1% BSA was added to the homogenate before being spun (800*g* for 15 min). The cell pellet was washed a further 2 × in PBS+0.1% BSA and a single-cell suspension was prepared for antibody staining. Fluorochrome-conjugated antibodies diluted 1/200 in PBS+0.1% BSA against the following antigens were used for flow cytometry: CD45 (30-F11; total leukocytes) and Ly6-C/G (RB6-8C5) were from BD Pharmingen; CD19 (eBio 1D3; B cells), TCRb (H57-597; T cells), F4/80 (BM8; macrophages), CD11c (N418; M1 macrophages), CD206 (C068C2; M2 macrophages) and CD115 (AFS98) were from eBiosciences. Flow cytometry was performed on a FACS Canto II running FACS Diva software. Representative FACS plots of CD45^+^ cells are shown in Supplementary Fig. 1 (WT and PKR KO fed SCD) and Supplementary Fig. 2 (WT and PKR KO fed HFD). In Supplementary Fig. 2, F4/80^+^ cells were gated for subsequent analysis of expression of CD206 and CD11c (middle two panels).

### Blood leukocytes

Whole blood was collected into tubes containing EDTA and immediately incubated on ice. All subsequent steps were performed on ice. Twenty microlitres of blood was used for total cell counting by an automated haematology analyser (XS-1000i, Sysmex Corporation). Red blood cells were lysed (BD pharm Lyse; BD Biosciences) and white blood cells centrifuged, washed and resuspended in HBSS (0.1% BSA (w/v), 5 mM EDTA). Cells were stained with appropriate fluorochrome-conjugated antibodies diluted 1/200. Monocytes were identified as CD45^hi^CD115^hi^, before being further subdivided into Ly6-C^hi^ and Ly6-C^lo^. Neutrophils were identified as CD45^hi^CD115^lo^Ly6-C/G (Gr1)^hi^. Samples were analysed on a FACS Canto II (BD Biosciences) and data were analysed using FlowJo software. The flow cytometry gating strategy we employed was as exactly as per that previously described in Nagareddy *et al*.^34^ Representative FACS plots of CD45^hi^ cells are shown in Supplementary Fig. 3.

### Gene expression

RNA was extracted from ∼50 mg of epididymal WAT using Trizol (Invitrogen) and total RNA measured using the ND-1000 NanoDrop Spectrophotometer (Thermo Scientific). RNA samples were treated with DNase I (Invitrogen) and cDNA generated using the Tetro cDNA synthesis kit (Bioline). Gene expression studies were performed by RT–PCR using TaqMan primers and probes for genes of interest and 18S rRNA (Applied Biosystems). The following TaqMan primer probe sets were used: *Emr1*—Mm00802530_m1; *Itgax*—Mm00498698_m1; *Tnf*—Mm00443258_m1; *Il1b*—Mm00434228_m1; *Ccl2*—Mm00441242_m1; *Cd3g*—Mm00438095_m1; *Ifng*—00801778_m1; *Il6*—Mm00446190_m1; *Elane*—Mm01168928_g1; 18S—4310893E. Twenty nanograms of cDNA was used per reaction. The comparative *C*_T_ method was used to quantify results from RT–PCR.

### Lipids

Plasma TAG (Roche Diagnostics), NEFA (NEFA-C kit; Wako Chemicals) and cholesterol (Wako) were determined enzymatically using colorimetric assays accordingly to the manufacturer's instructions. Liver TAG were assessed following the extraction of total lipids from ∼50 mg of the liver. Briefly, liver was homogenized in 2:1 chloroform/methanol and shaken for 1 h at room temperature. Following the addition of H_2_O samples were spun (2,000 r.p.m. for 10 min), the lower chloroform phase collected, dried under nitrogen and the extract dissolved in ethanol before the determination of TAG concentrations (Roche Diagnostics).

### Genotyping

Mice were initially genotyped at 3 weeks of age and assigned to appropriate groups. Following completion of the dietary period we re-genotyped all animals to confirm mice had been assigned to the correct groups. Genotyping of tail samples was performed using a forward primer common to both the WT and mutated *Eif2ak2* allele (5′- GTGGTGGGTTGGAAACACCAGATCTGATG -3′) and reverse primers specific for the WT (5′- CCTGGCCCTAACTACATCTTTCCTGATG -3′) and mutated (PKR KO; 5′- CCCGATTCGCAGCGCATCGCCTTCTATC -3′) *Eif2ak2* alleles. DNA was extracted from mouse tails using the MyTaq Extract-PCR kit (Bioline) according to the manufacturer's instructions. The following cycling conditions were used: 1 cycle of 3 min at 95 °C followed by 35 cycles of 15 s at 95 °C, 15 s at 70 °C, 20 s at 72 °C followed by a final step at 72 °C for 5 min. Products were resolved on a 1% agarose gel containing ethidium bromide.

### Sample sizes and exclusion of mice

Power calculations were not performed. Sample sizes were based on experimental feasibility as well as prior knowledge of statistical power from our own experience and previously published work. At the start of the study, 9, 8, 12 and 18 animals were assigned to WT SCD, PKR KO SCD, WT HFD and PKR KO HFD, respectively. Exclusion of mice: In Fig. 2h, one mouse was excluded from the PKR KO HFD group as both the 0 and 15 min samples were greater than 3s.d. from the group mean. In Fig. 4b,d,e, only a subset of animals was used for flow cytometry and no data were excluded from this analysis. In Fig. 4a, one mouse from the PKR KO SCD was excluded because of poor RNA yield following RNA isolation. In Fig. 4c, four mice were excluded from the PKR KO HFD group because of poor RNA yield following RNA isolation. An additional 1–3 mice were excluded from the PKR KO HFD group in Fig. 4c where the expression of the ‘housekeeping' gene 18S was greater than 3 s.d. from the mean.

### Statistical analysis

Statistical analysis was conducted using IBM SPSS Statistics Version 22. Statistical significance was calculated by independent samples *t*-test, two-way ANOVA or two-way repeated-measures ANOVA as appropriate. Gene expression data were log transformed before statistical analysis because it was abnormally distributed. Data throughout the paper are shown as mean±the standard deviation (s.d.). A *P* value of less than 0.05 was considered statistically significant.

## Additional information

**How to cite this article**: Lancaster, G. I. *et al*. PKR is not obligatory for high-fat diet-induced obesity and its associated metabolic and inflammatory complications. *Nat. Commun.* 7:10626 doi: 10.1038/ncomms10626 (2016).

## Supplementary Material

Supplementary Information Supplementary Figures 1-5
