# Supplementary material for: PKR is not obligatory for high-fat diet-induced obesity and its associated metabolic and inflammatory complications
Source: Nat Commun. 2016 Feb 3;7:10626. doi: 10.1038/ncomms10626 (PMC4743083; doi:10.1038/ncomms10626)
Supplement: Supplementary Information — Supplementary Figures 1-5 [file ncomms10626-s1.pdf]

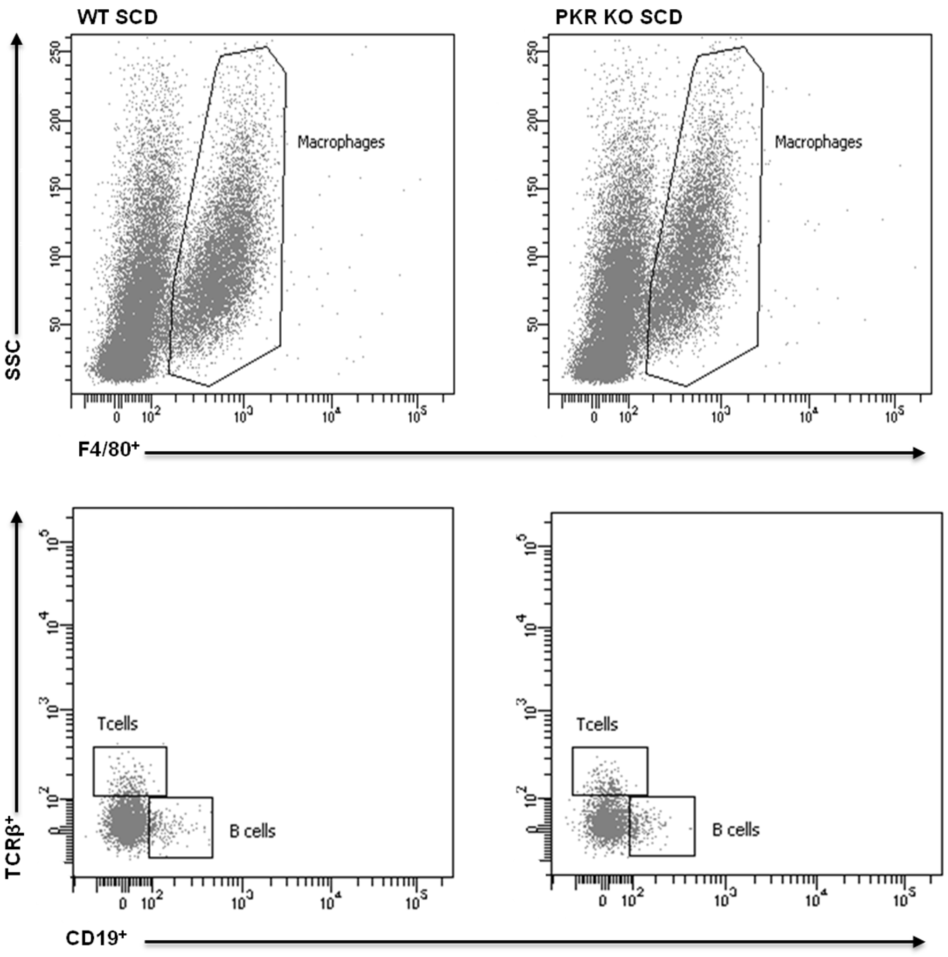

Supplementary Figure 1
Representative FACS plots of the data shown in Figure 4b.

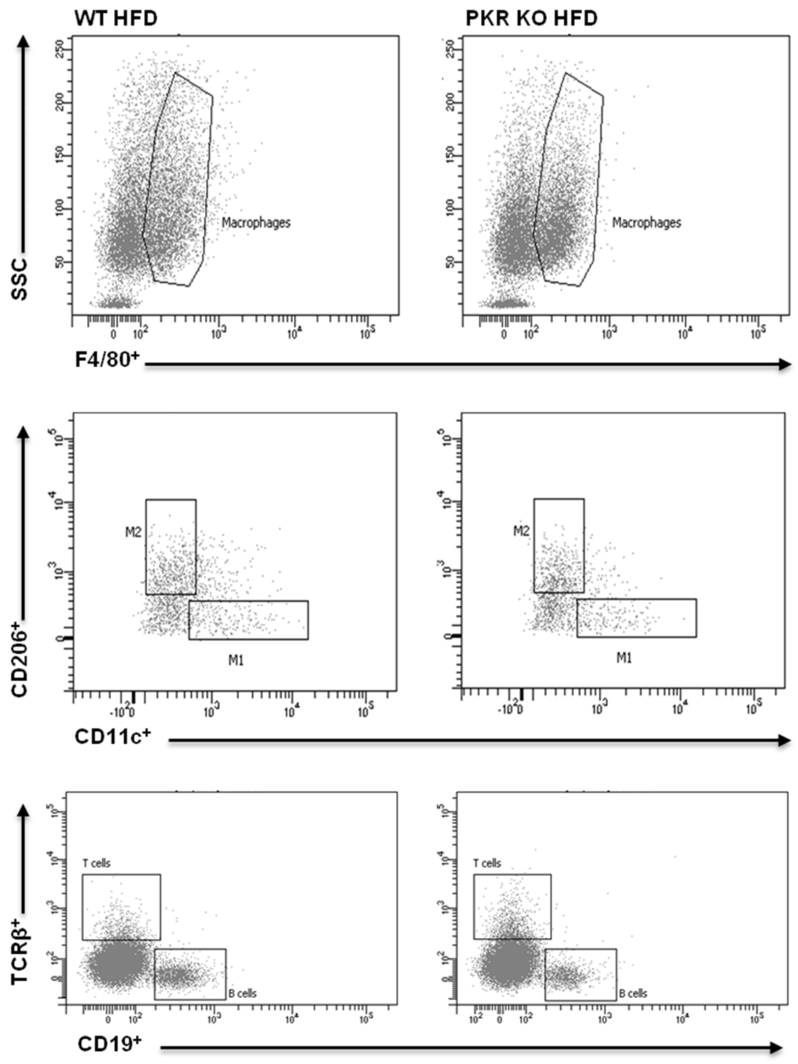

Supplementary Figure 2

Representative FACS plots of the data shown in Figure 4d,e.

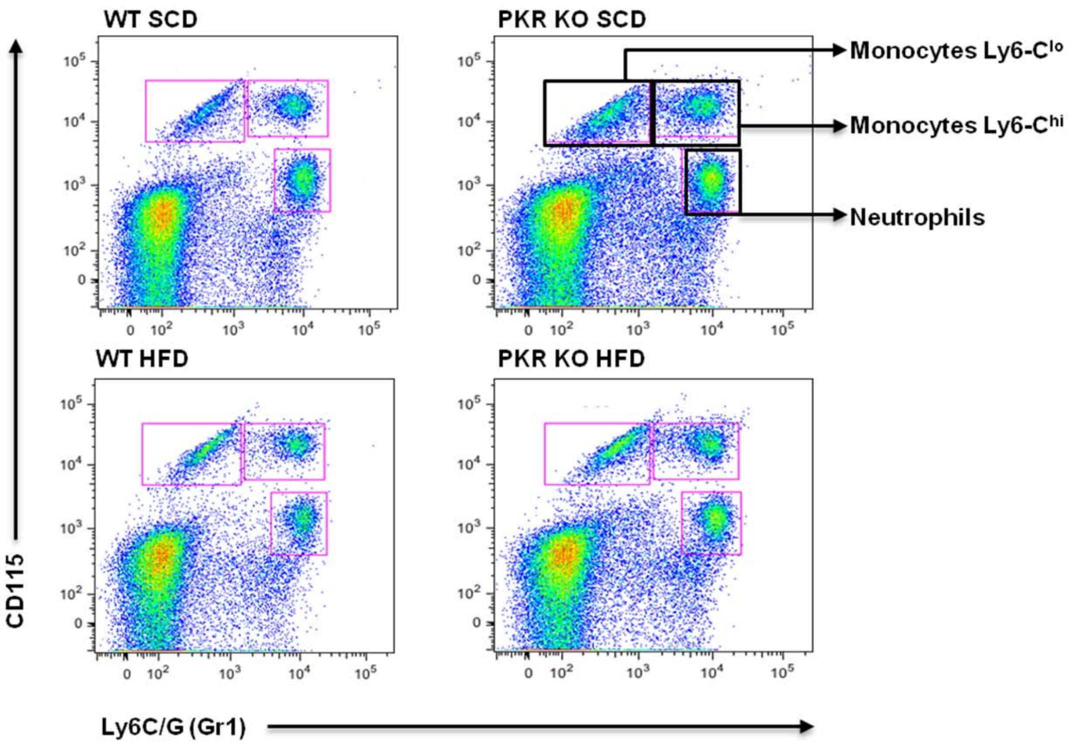

Supplementary Figure 3
Representative FACS plots of the data shown in Figure 4f.

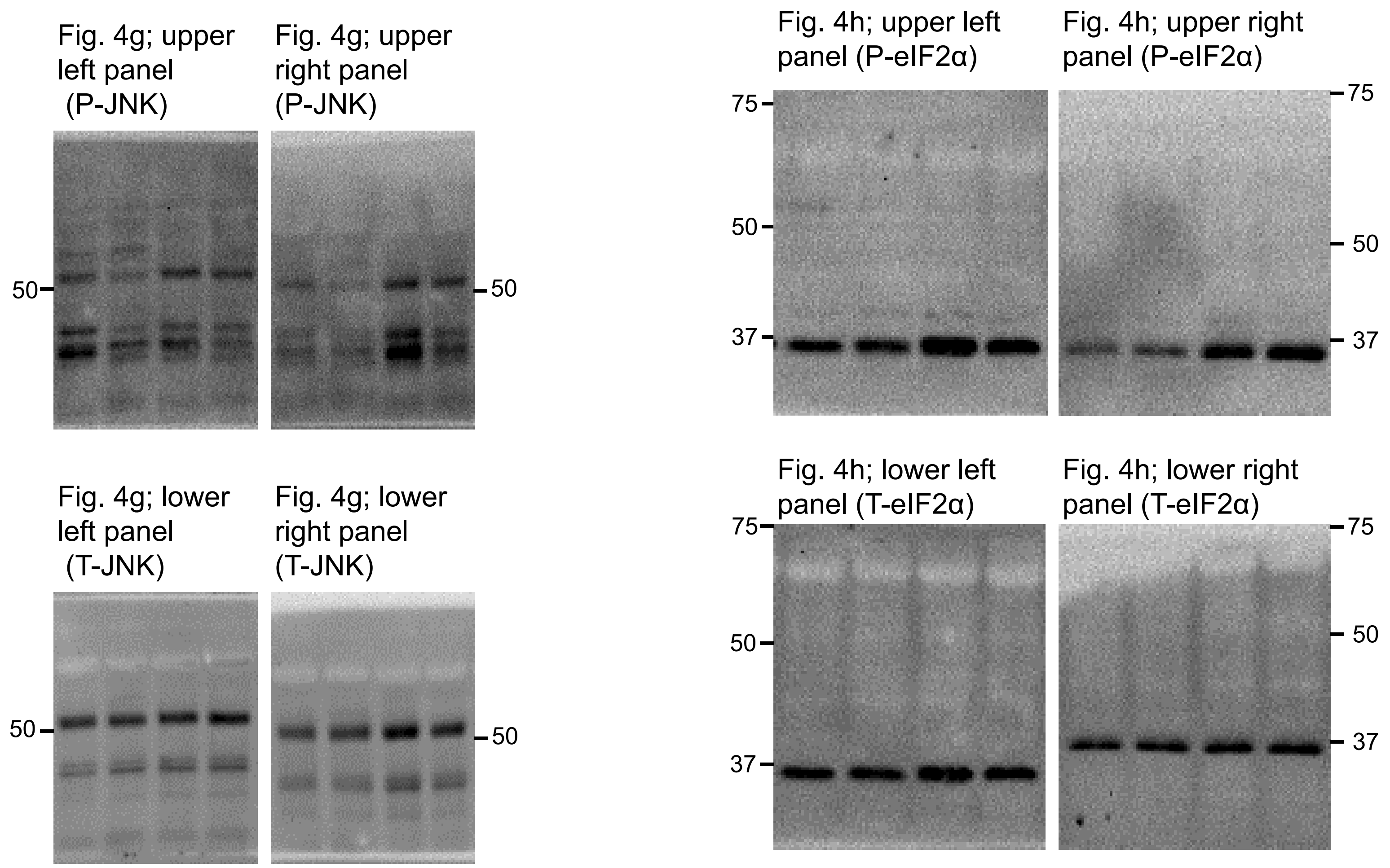

Supplementary Figure 4

Uncropped images of the blots shown in Fig. 4g,h.

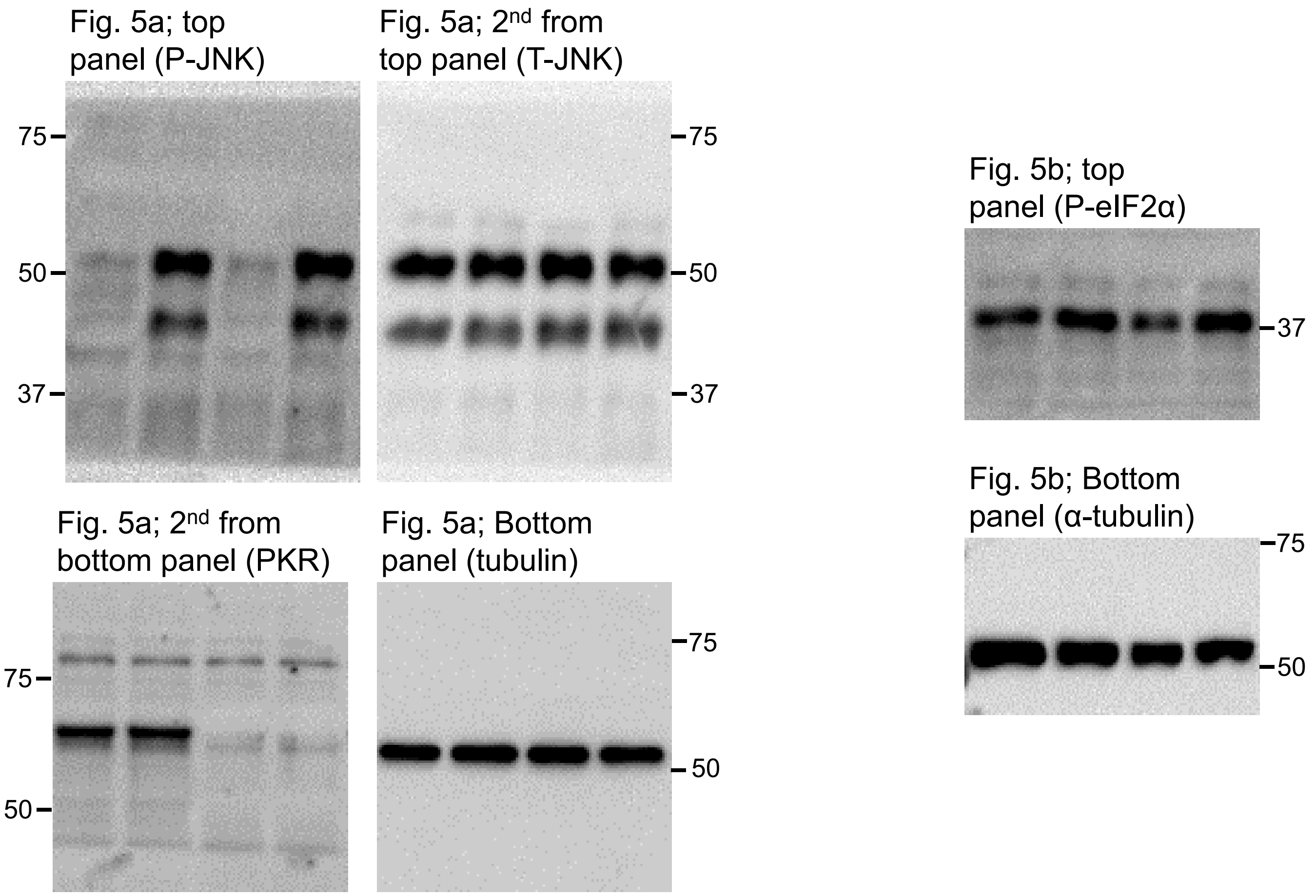

Supplementary Figure 5
Uncropped images of the blots shown in Fig. 5a,b.
